# Are physical inactivity, sitting time and screen time associated with obstructive sleep apnea in adults? A cross-sectional study

**DOI:** 10.1590/1516-3180.2020.0651.R2.08062021

**Published:** 2022-02-21

**Authors:** Rafael Mathias Pitta, Bruno Gion Cerazi, Luana Queiroga, Raphael Mendes Ritti Dias, Marco Túlio de Mello, Fernando Henpin Yue Cesena, Roberta Luksevicius Rica, Julien Steven Baker, Marcio Sommer, Gabriel Grizzo Cucato, Danilo Sales Bocalini, Oskar Kauffman

**Affiliations:** I MSc. Professor, Sciences of Aging, Hospital Israelita Albert Einstein (HIAE), São Paulo (SP), Brazil.; II MSc. Professor, Sciences of Aging, Hospital Israelita Albert Einstein (HIAE), São Paulo (SP), Brazil.; III MSc. Professor, Sciences of Aging, Hospital Israelita Albert Einstein (HIAE), São Paulo (SP), Brazil.; IV PhD. Professor, Rehabilitation Sciences, Universidade Nove de Julho, São Paulo (SP), Brazil.; V PhD. Professor, Sports Department, Universidade Federal de Minas Gerais (UFMG), Belo Horizonte (MG), Brazil.; VI MD, PhD. Attending Physician, Sciences of Aging, Hospital Israelita Albert Einstein (HIAE), São Paulo (SP), Brazil.; VII MSc, PhD. Professor, Department of Physical Education, Estácio de Sá University, Vitória (ES), Brazil.; VIII PhD, DSc. Professor, Department of Sport, and Physical Education, Hong Kong Baptist University, Kowloon Tong, Hong Kong, China.; IX MD, PhD. Attending Physician, Sciences of aging, Hospital Israelita Albert Einstein (HIAE), São Paulo (SP), Brazil.; X PhD. Professor, Department of Sport, Exercise & Rehabilitation, Northumbria University, Newcastle Upon Tyne, United Kingdom.; XI PhD. Professor, Physical Education and Sports Center, Universidade Federal do Espírito Santo (UFES), Vitória, ES, Brazil.; XII MD, PhD. Attending Physician, Sciences of Aging, Hospital Israelita Albert Einstein (HIAE), São Paulo (SP), Brazil.

**Keywords:** Obesity, Sleep apnea, obstructive, Exercise, Sedentary behavior, Polysomnography, Sleep apnea, Physical activity, Sedentary lifestyle, Sleep monitoring, Sedentary time

## Abstract

**BACKGROUND:**

Sitting time, screen time and low physical activity (PA) levels have been associated with several diseases and all-cause mortality. PA is related to better sleep quality and absence of daytime sleepiness, along with lower risks of obstructive syndrome apnea (OSA). However, studies on the relationship between sitting time, screen time and OSA are scarce in the literature.

**OBJECTIVE:**

To analyze associations between PA levels, sitting time, screen time and OSA among adults with suspected sleep disorder.

**DESIGN AND SETTING:**

Cross-sectional study conducted at Hospital Israelita Albert Einstein.

**METHODS:**

Data were collected from 369 adults with suspected sleep disorders who visited the hospital’s neurophysiology clinic between August 2015 and January 2017.

**RESULTS:**

Correlations between hypopnea and PA indicators were demonstrated for total sitting time (0.123; P = 0.019) and total screen time (0.108; P = 0.038). There was also a correlation between latency for rapid-eye-movement sleep (REM_LAT) and total sitting time (0.103; P = 0.047) and a negative correlation between mean oxyhemoglobin saturation (SaO_Avg) and total PA time (-0.103; P = 0.048). There were no associations between PA parameters and apnea-hypopnea index. After adjusting for confounding factors (body mass index, age and gender), sitting time and screen time were not associated with OSA.

**CONCLUSION:**

After adjusting for anthropometric and clinical factors, excessive sitting time or screen time was not associated with OSA in adults suspected of sleep disorders. Age, gender, hypertension, body mass index and waist circumference were associated with OSA.

## INTRODUCTION

Obstructive syndrome apnea (OSA) is characterized by repetitive collapse of the upper airways during sleep and is defined by an apnea-hypopnea index (AHI) ≥ 15 events / hour, with reduced airflow, oxygen desaturation and sleep interruption.^1^ OSA is associated with chronic diseases such as hypertension, metabolic comorbidities and increased all-cause mortality.^2,3,4,5^

The potential serious adverse consequences of untreated OSA are a major reason for emphasizing early diagnosis and treatment.^6,7^ The main risk factors for OSA are obesity, age, cranial issues and gender (men).^6,8,9^ Moreover physical activity (PA) levels and sedentary behavior (SED, characterized as sitting time, screen time and low energy expenditure)^10^ have been identified as new risk factors for most chronic diseases, such as cardiovascular disease, diabetes and some cancers.^11,12,13^ There is evidence demonstrating associations between these risk factors and OSA.^14^

Although previous studies have explored the association between OSA, PA and SED, no study has analyzed these associations using overnight polysomnography (PSG) as a method for diagnosing OSA.

## OBJECTIVE

The purpose of this study was to analyze associations between PA levels, SED and OSA among adults with suspected sleep disorders that were diagnosed at a neurophysiology laboratory for OSA.

## METHODS

This study had a cross-sectional design. The ethics committee of Hospital Israelita Albert Einstein approved the study protocol (SGPP: 1.150.084/2015 and CAAE: 45354215.4.0000.0071; date: July 15, 2015). Subsequently, individuals who visited the neurophysiology clinic at this hospital between August 2015 and January 2017 were invited to participate and signed a written consent statement in order to participate. The following inclusion criteria for participation were adopted: age over 18 years and indication for undergoing overnight laboratory PSG due to suspected OSA. Patients were excluded if they were already receiving treatment for OSA, had a disease that would make it impossible to complete the questionnaires or had technical problems during the overnight laboratory PSG.

### Overnight laboratory polysomnography

All individuals participated in an overnight laboratory PSG as previously described.^15,16,17^ Individuals were prepared between 8:30 pm and 10:30 pm and were woken up between 6:00 am and 7:00 am, when the recording was ended. The following signals were recorded: electroencephalograms (C3M2, C4M1 and O2M1), bilateral electrooculograms, electromyograms of the chin muscles and right and left anterior tibialis, movement of the rib cage and abdomen (piezoelectric crystal), oxygen saturation (SaO_2_) from pulse oximetry, electrocardiogram (lead 1) and body position. Airflow was assessed from nasal airway pressure and oronasal thermistry. Trained PSG technicians, blinded to the study, positioned the patients and monitored them throughout the night, as recommended in the American Academy of Sleep Medicine Manual.^10^ Apnea events were defined as ≥ 90% airflow reduction for ≥ 10 seconds. Hypopnea events were defined as ≥ 30% airflow reduction in association with ≥ 3% drop in oxygen desaturation or sleep fragmentation. To classify the presence and severity of OSA, the cutoff points for apnea were used: ≥ 15 and < 30 events/hour (moderate apnea); and ≥ 30 events/hour (severe apnea).^18,19^

### Physical activity and sedentary behavior

To analyze PA levels and SED, we used the International Physical Activity Questionnaire (IPAQ),^20^ which evaluates frequency, intensity and duration of physical activity, classifying individuals into four categories (very active, active, non-active and sedentary), as well as evaluating the total sitting time and screen time per week and at weekends.^21,22,23,24^ Cutoff scores were used for sitting and screen time using different models (model 1: < 6 and ≥ 6 hour/day; model 2: < 10 and ≥ 10 hour/day) and different PA levels, according to the criteria below:

a- Active individuals, in line with the following recommendations for PA: (1): a) vigorous: ≥ 5 days / week and ≥ 30 minutes per session and/or b) vigorous: ≥ 3 days/week and ≥ 20 minutes per session + moderate PA and/or c) walking: ≥ 5 days / week and ≥ 30 minutes per session; or (2): a) vigorous: ≥ 3 days/week and ≥ 20 minutes per session and/or b) moderate or walking: ≥ 5 days / week and ≥ 30 minutes per session and/or any activity accumulated: ≥ 5 days / week and ≥ 150 minutes/week (walking + moderate +vigorous);

b- Inactive individuals, who did PA but insufficiently to be classified as active because they did not comply with the recommendations regarding frequency or duration. In this classification, we included the frequency and duration of different types of activities (vigorous + moderate + walking). Thus, individuals were considered inactive if they met at least one of the criteria of the recommendation regarding frequency or duration of the activity: a) frequency: 5 days/ week or b) duration: 149 minutes/week; or if they failed to perform continuous physical activity for at least 10 minutes during the week.

### Anthropometric and study covariates

The characteristics of interest included age, gender, body mass index (BMI), waist circumference and presence of hypertension (HTN). Anthropometric assessments of height, body weight and abdominal circumference were made in accordance with standard procedures.

Body weight was determined by using the InBody 270 scale (Ottoboni, Rio de Janeiro, Brazil). Individuals positioned themselves on this calibrated scale by placing their feet (without socks or shoes) on the metal region of the platform, with feet facing forward. Height was determined using a stadiometer with an accuracy of 0.1 mm. Individuals were positioned with their heels, gluteal scapulas and occipital surfaces in contact with the wall. During the measurement, the individuals performed inspiratory apnea, looking to the horizon, and at that point the evaluator placed the cursor of the stadiometer on the apex of the head. The measurement was performed three times and the final result was the mean of the three measurements.

From these measurements, the individuals’ BMI was calculated. The classification adopted for BMI followed the criteria outlined by the World Health Organization for adults: eutrophic (18.5-24.9 kg/m^2^); overweight (25.0-29.9 kg/m^2^); and obese (≥ 30.0 kg/m^2^).

For waist circumference (WC) measurements, individuals remained in the orthostatic position, with a relaxed abdomen. A tape measure was positioned in the horizontal plane at the midpoint between the last costal arch and the iliac crest. Measurements were made three times and the final result was the mean of the three measurements. The cutoff point for waist circumference measurements was in accordance with the World Health Organization guidelines for adults: men ≥ 94 cm and women ≥ 80 cm; and for elderly people: men ≥ 102 cm and women ≥ 88 cm.

Blood pressure was measured in accordance with international standards, using an aneroid sphygmomanometer with a Dormed pedestal (Dormed, Belo Horizonte, Minas Gerais, Brazil). In the supine position, the arms were kept alongside the body, one of them with a slightly abducted cuff; for patients with an extremely developed thorax, cushions were used to secure the arm at the level of the heart. HTN was considered present when the blood pressure was ≥ 140/90 mmHg. In addition, patients with a history of HTN and patients who were using antihypertensive medications were considered to have HTN.

### Statistical analyses

Multinominal models were constructed for each explanatory variable and for each outcome. The odds ratio (OR) was calculated with the respective confidence interval and P-value.

The first model compared moderate (15 ≥ AHI ≥ 30) with absent apnea (< 15 AHI), and the second model compared severe (≥ 30 AHI) with absent apnea. Pearson’s correlation coefficients were used to evaluate the relationships between weekly PA indicators (total physical activity time, total sitting time and total screen time) and sleeping indicators monitored by means of PSG. In addition, groups formed by the combination of PA time (active or inactive) with screen time (short or long time) were compared with sleep indicators obtained by means of PSG, using generalized linear models. Different probability and bond function distributions were tested, and the best-fit model was chosen in accordance with the AIC44 fit quality criterion.

The results were presented as mean values estimated through the models at 95% confidence intervals. In comparing the groups formed by the combination of PA time (active or inactive) with sitting time (short or long time), the same procedure was used for the analysis as was used for groups investigating screen time. Multiple logistic regression models were built to investigate the relationship between PA level and SED, controlling for age, gender and BMI.

All the analyses were done using the Statistical Package for the Social Sciences (SPSS) software, version 21 (IBM, SPSS Inc., Chicago, United States),^25^ and the significance level used was 5%.

## RESULTS

Table 1 shows the demographic data of the sample, together with the ORs of the variables in relation to OSA severity. The sample was characterized as middle-aged (between 34 and 56 years old); 66.94% were men; and there was high prevalence of overweight or obesity in OSA presence (between 25.88 and 34.58 kg/m^2^). The diagnosis of HTN was observed in 16.29% of participants without OSA, 26.44% of those with moderate OSA and 30.77% of those with severe OSA, respectively. Clinical characteristics such as male gender, obesity, elevated WC and HTN presence were associated with moderate and severe OSA among individuals with suspected sleep disorders.

**Table 1.** Association between obstructive syndrome apnea severity obtained from polysomnography and anthropometric factors

Variable	Presence of obstructive syndrome apnea			OR [95% CI (moderate to absent)]	P-value	OR [95% CI (severe to absent)]	P-value
	AHI < 15	15 ≥ AHI < 30	AHI ≥ 30				
Age (years)							
Mean (SD)	42.05 (11,95)	46.94 (11.18)	48.67 (12)	1.04 [1.01; 1.06]	0.002	1.05 [1.03; 1.07]	< 0.001
Median [Q1; Q3]	42.00 [34.00; 49.75]	47.00 [39.50; 54.00]	48.00 [39.00; 56.25]				
N	178	87	104				
Gender							
Women	84 (47.19%)	19 (21.84%)	19 (18.27%)	1 (Reference)		1 (Reference)	
Men	94 (52.81%)	68 (78.16%)	85 (81.73%)	3.20 [1.78; 5.76]	< 0.001	3.99 [2.24; 7.12]	< 0.001
N	178	87	104				
BMI (kg/m^2^)							
Mean (SD)	26.72 (4.41)	28.95 (4.66)	31.25 (5.22)	1.12 [1.05; 1.19]	< 0.001	1.22 [1.15; 1.29]	< 0.001
Median [Q1; Q3]	26.45 [23.38; 29.12]	28.40 [25.88; 30.79]	30.70 [27.67; 34.58]				
N	178	87	104				
WC							
0	48 (30.97%)	13 (19.4%)	8 (9.76%)	1 (Reference)		1 (Reference)	
1	107 (69.03%)	54 (80.6%)	74 (90.24%)	1.86 [0.93; 3.73]	0.08	4.15 [1.85; 9.28]	0.001
N	155	67	82				
Hypertension							
Absence	149 (83.71%)	64 (73.56%)	72 (69.23%)	1 (Reference)		1 (Reference)	
Presence	29 (16.29%)	23 (26.44%)	32 (30.77%)	1.85 [0.99; 3.43]	0.05	2.28 [1.28; 4.06]	0.005
N	178	87	104				

AHI = apnea-hypopnea index (events per hour); BMI = body mass index; WC = waist circumference; CI = confidence interval; OR: odds ratio. Waist circumference category 0: men < 94 cm and women < 80 cm; elderly men: < 102 cm and elderly women: < 88 cm. Waist circumference category 1: men ≥ 94 cm and women ≥ 80 cm; elderly men: ≥ 102 cm and elderly women: ≥ 88 cm. Data are presented as mean, median and standard deviation.

The correlation between PA and SED indicators (total physical activity time, sitting time and screen time) and sleep indicators from PSG are shown in Table 2. The correlation coefficients obtained indicated that there was a low correlation between REM_LAT and total sitting time (r = 0.103; P = 0.047) and a low negative correlation between SaO_Avg and total PA time (r = -0.103; P = 0.048). In addition, no correlations were found between AHI and total physical activity time (r = 0.084, P = 0.107), total sitting time (r = 0.067, P = 0.202) or total screen time (r = 0.036, P = 0.485).

**Table 2.** Correlation between coefficients of measurements of physical activity indicators (total physical activity time, total sitting time and total screen time) and sleep quality indicators obtained from polysomnography, among patients who underwent the examination

Polysomnography	TAFT	P	TST	p	TTT	P
TTR	-0.062	0.237	0.003	0.960	0.038	0.473
SLEEP_LAT	-0.049	0.350	-0.043	0.409	0.035	0.498
REM_LAT	-0.057	0.278	0.103	0.047*	0.013	0.810
TTS	-0.013	0.805	0.035	0.504	0.036	0.488
%NI	-0.054	0.302	0.046	0.378	0.033	0.522
%NII	0.018	0.736	0.026	0.613	0.058	0.268
%NIII	0.005	0.931	-0.033	0.529	-0.064	0.223
%REM	0.037	0.478	-0.042	0.421	-0.035	0.504
SLEEP_WAKE	-0.028	0.593	0.001	0.987	-0.057	0.272
NARO	-0.094	0.073	0.072	0.169	0.034	0.521
IND_NARO	-0.097	0.063	0.047	0.369	0.049	0.351
RESP_BREAKS	-0.079	0.130	0.089	0.088	0.045	0.386
APNEA	-0.069	0.185	0.032	0.544	-0.022	0.667
OBS_APNEA	-0.080	0.126	0.048	0.361	-0.007	0.896
CENT_APNEA	-0.006	0.904	-0.063	0.227	-0.051	0.330
MIX_APNEA	-0.001	0.978	-0.010	0.855	-0.016	0.766
HYPOPNEA	-0.075	0.151	0.123	0.019*	0.108	0.038*
SaO_Avg	-0.103	0.048*	0.021	0.689	0.063	0.227
SaO_Min	0.066	0.208	-0.074	0.155	0.039	0.459
%SAO	-0.042	0.418	-0.016	0.755	-0.042	0.420
AHI	-0.084	0.107	0.067	0.202	0.036	0.485

Data are shown as Pearson correlation coefficients. TAFT = total physical activity time; TST = total sitting time; TTT = total screen time; TTR = total recording time; SLEEP_LAT = sleep latency; REM_LAT = REM sleep latency; TTS = total sleep time; %NI = percentage sleep stage 1; %NII = percentage sleep stage 2; %NIII = percentage sleep stage III; %REM = percentage REM sleep; SLEEP_WAKE = awakening from sleep; NARO = nightly arousals; IND_NARO = nightly arousals index; RESP_BREAKS = respiratory breaks; OBS_APNEA = obstructive apnea; CENT_APNEA = central apnea; MIX_APNEA = mixed apnea; HYPOPNEA = hypopnea; SaO_Avg = average oxygen saturation; SaO_Min = minimum oxygen saturation; %SAO = percentage oxygen apnea; AHI = apnea-hypopnea index (events per hour). *P < 0.05.

### Screen time and physical activity

Comparisons of sleep indicators obtained from PSG in relation to PA level and screen time are shown in Table 3. For this evaluation, four groups were created, combining the levels of PA: active (≥ 150 minutes) or inactive (< 150 minutes) with short screen time (< 6 hours and < 10 hours) or long screen time (≥ 6 hours and ≥ 10 hours). We observed differences between the groups in terms of percentage REM sleep (%REM; P = 0.030) and awakening from sleep (SLEEP_WAKE, P = 0.028). In relation to %REM, the average for the inactive group with short screen time was lower than the averages for the inactive group with long screen time (P = 0.019) and the active group with long screen time (P = 0.032). In relation to SLEEP_WAKE, the average for the inactive group with short screen time was higher than the average for the active group with short screen time (P = 0.044). Moreover, we did not see any evidence of differences between PA indicators in PSG measurements (P > 0.05 in all comparisons).

**Table 3.** Estimated average values and 95% confidence intervals for sleep quality indicators obtained from polysomnography, among patients undergoing the examination according to physical activity and screen time

PSG	Physical activity time and screen time (< 6 h or ≥ 6 h)					Physical activity time and screen time (< 10 h or ≥ 10 h)				
	Inactive with short screen time (n = 31)	Inactive with long screen time (n = 135)	Active with short screen time (n = 32)	Active with long screen time (n = 171)	P-value	Inactive with short screen time (n = 65)	Inactive with long screen time (n = 101)	Active with short screen time (n = 91)	Active with long screen time (n = 112)	P-value
TTR	449.4 (433.7; 464.3)	442.2 (434.6; 449.6)	437.4 (421.4; 452.6)	439.5 (432.7; 446.2)	0.654	448.8 (438.0; 459.1)	440.2 (431.4; 448.8)	439.3 (429.9; 448.3)	439.1 (430.7; 447.3)	0.497
SLEEP_LAT	22.8 (14.0; 37.2)	23.6 (18.7; 29.8)	16.3 (10.1; 26.3)	17.6 (14.3; 21.7)	0.230	20.5 (14.7; 28.8)	25.5 (19.4; 33.3)	17.2 (12.9; 22.9)	17.6 (13.6; 22.8)	0.165
REM_LAT	111.5 (77.0; 161.3)	99.6 (83.5; 119.0)	118.6 (82.4; 170.7)	106.3 (90.8; 124.4)	0.828	104.3 (80.8; 134.6)	100.2 (81.6; 123.0)	116.2 (93.7; 144.2)	101.8 (83.8; 123.7)	0.765
TTS	350.3 (327.7; 371.3)	367.2 (357.2; 376.9)	376.0 (355.7; 394.7)	371.6 (362.9; 380.0)	0.265	365.7 (351.1; 379.6)	363.1 (351.3; 374.4)	369.4 (357.2; 381.0)	374.6 (363.9; 384.9)	0.513
%NI	10.1 (8.0; 12.8)	9.6 (8.6; 10.7)	8.6 (6.8; 10.7)	9.5 (8.6; 10.5)	0.774	9.3 (7.9; 10.9)	9.9 (8.7; 11.3)	9.3 (8.1; 10.7)	9.4 (8.3; 10.6)	0.897
%NII	56.8 (53.4; 60.3)	53.9 (52.3; 55.5)	57.5 (54.2; 61.1)	53.8 (52.4; 55.2)	0.095	54.0 (51.7; 56.2)	54.7 (52.9; 56.5)	53.9 (52.0; 55.8)	54.7 (53.0; 56.5)	0.881
%NIII	16.9 (13.9; 19.9)	16.5 (15.1; 18.0)	15.2 (12.2; 18.1)	17.4 (16.1; 18.7)	0.540	17.9 (15.8; 20.0)	15.8 (14.1; 17.4)	17.3 (15.6; 19.1)	16.8 (15.3; 18.4)	0.403
%REM	14.7 (12.3; 17.1)	18.7 (17.5; 19.8)	18.2 (15.9; 20.6)	18.3 (17.3; 19.3)	0.030*	17.7 (16.0; 19.4)	18.1 (16.7; 19.4)	18.7 (17.3; 20.1)	17.9 (16.7; 19.2)	0.810
SLEEP_WAKE	62.4 (47.8; 81.5)	41.4 (36.5; 47.1)	37.1 (28.6; 48.3)	44.2 (39.5; 49.5)	0.028*	50.0 (41.5; 60.2)	42.0 (36.2; 48.7)	44.9 (38.3; 52.5)	41.6 (36.1; 47.9)	0.412
NARO	118.9 (92.9; 152.2)	113.3 (100.6; 127.5)	100.0 (78.4; 127.5)	113.3 (102.0; 125.9)	0.770	113.5 (95.7; 134.6)	114.8 (100.1; 131.7)	105.4 (91.3; 121.7)	115.9 (101.8; 132.0)	0.780
IND_NARO	21.2 (16.3; 26.7)	20.3 (18.0; 22.9)	17.0 (12.7; 21.9)	18.8 (16.8; 21.0)	0.513	19.3 (16.0; 22.9)	21.3 (18.5; 24.3)	17.8 (15.1; 20.7)	19.1 (16.6; 21.8)	0.397
RESP_BREAKS	130.1 (85.4; 198.2)	113.5 (92.8; 138.9)	76.0 (50.2; 115.0)	105.8 (88.4; 126.6)	0.285	121.0 (90.5; 161.9)	113.7 (90.0; 143.6)	88.1 (68.9; 112.6)	111.7 (89.5; 139.4)	0.321
APNEA	3.8 (2.0; 6.5)	2.4 (1.7; 3.2)	3.0 (1.5; 5.2)	2.5 (1.9; 3.3)	0.566	3.0 (1.9; 4.4)	2.4 (1.6; 3.3)	2.5 (1.7; 3.6)	2.7 (1.9; 3.7)	0.882
OBS_APNEA	2.1 (1.0; 3.9)	1.2 (0.8; 1.7)	1.3 (0.5; 2.6)	1.3 (0.9; 1.8)	0.584	1.8 (1.2; 2.9)	1.1 (0.8; 1.5)	1.4 (1.0; 2.0)	1.3 (0.9; 1.7)	0.349
CENT_APNEA	0.67 (0.25; 1.23)	0.51 (0.31; 0.73)	0.70 (0.28; 1.26)	0.59 (0.40; 0.80)	0.838	0.56 (0.27; 0.90)	0.52 (0.29; 0.78)	0.52 (0.28; 0.80)	0.68 (0.44; 0.96)	0.786
MIX_APNEA	0.34 (0.05; 0.72)	0.23 (0.09; 0.39)	0.29 (0.01; 0.65)	0.28 (0.15; 0.42)	0.924	0.32 (0.11; 0.56)	0.21 (0.06; 0.39)	0.30 (0.12; 0.50)	0.27 (0.11; 0.45)	0.883
HYPOPNEA	88.1 (61.1; 126.9)	90.4 (75.9; 107.6)	56.9 (39.6; 81.6)	82.3 (70.4; 96.1)	0.155	86.1 (66.8; 110.8)	92.5 (75.5; 113.2)	69.6 (56.2; 86.2)	85.1 (70.2; 103.1)	0.278
SaO_Avg	93.4 (92.5; 94.1)	93.4 (93.0; 93.8)	93.7 (93.0; 94.5)	93.5 (93.2; 93.9)	0.837	93.2 (92.6; 93.7)	93.5 (93.1; 94.0)	93.5 (93.0; 93.9)	93.6 (93.2; 94.0)	0.589
SaO_Min	83.0 (80.3; 85.4)	83.9 (82.7; 85.1)	84.7 (82.2; 87.0)	84.4 (83.3; 85.4)	0.708	83.0 (81.2; 84.7)	84.2 (82.8; 85.5)	84.7 (83.3; 86.1)	84.2 (82.9; 85.4)	0.489
%SAO	1.6 (0.8; 2.6)	0.8 (0.5; 1.1)	0.5 (0.1; 1.1)	0.8 (0.6; 1.1)	0.131	1.2 (0.7; 1.7)	0.8 (0.5; 1.2)	0.7 (0.4; 1.1)	0.8 (0.5; 1.2)	0.494
AHI	23.0 (16.0; 31.1)	20.5 (17.2; 24.1)	14.2 (8.8; 20.7)	17.9 (15.1; 20.8)	0.195	20.8 (16.1; 26.1)	21.1 (17.2; 25.3)	15.6 (12.1; 19.5)	18.7 (15.2; 22.5)	0.199

Data are presented as estimated averages (95% CI); active (≥ 150 minutes) or inactive (<150 minutes); short time (<6 hours and < 10 hours) or long time (≥ 6 hours and ≥ 10 hours). TAFT = total physical activity time; TST = total sitting time; TTT = total screen time; TTR = total recording time; SLEEP_LAT = sleep latency; REM_LAT = REM sleep latency; TTS = total sleep time; %NI = percentage sleep stage 1; %NII = percentage sleep stage 2; %NIII = percentage sleep stage III; %REM = percentage REM sleep; SLEEP_WAKE = awakening from sleep; NARO = nightly arousals; IND_NARO = nightly arousals index; RESP_BREAKS = respiratory breaks; OBS_APNEA = obstructive apnea; CENT_APNEA = central apnea; MIX_APNEA = mixed apnea; HYPOPNEA = hypopnea; SaO_Avg = average oxygen saturation; SaO_Min = minimum oxygen saturation; %SAO = percentage oxygen apnea, AHI: apnea-hypopnea index. *P < 0.05.

### Sitting time and physical activity

Table 4 shows the comparisons between sleep indicators obtained from PSG in relation to the levels of PA and sitting time using the same combinations of screen time. Using the 6-hour cut-off time it was not possible to adjust the models to compare these groups. In the adjusted models for comparing the PA and sitting time group, considering the 10-hour cutoff, no evidence of differences in PSG measurements (P > 0.05 in all comparisons) were found between the groups.

**Table 4.** Estimated average values and 95% confidence intervals for sleep quality indicators obtained from polysomnography, among patients who underwent the examination, according to physical activity (active or inactive) and sitting time (< 10 hours or ≥ 10 hours).

Polysomnography	Physical activity group and sitting time				P-value
	Inactive with short sitting time (n = 41)	Inactive with long sitting time (n = 125)	Active with short sitting time (n = 40)	Active with long sitting time (n = 163)	
TTR	451.7 (438.2; 464.6)	440.9 (433.0; 448.6)	438.2 (423.9; 451.8)	439.4 (432.5; 446.2)	0.421
SLEEP_LAT	25.0 (16.4; 38.2)	23.0 (18.0; 29.3)	20.4 (13.3; 31.4)	16.7 (13.5; 20.7)	0.170
REM_LAT	100.6 (72.9; 138.8)	102.2 (85.0; 122.9)	103.9 (75.0; 143.9)	109.3 (93.0; 128.4)	0.943
TTS	356.8 (337.7; 374.8)	366.5 (356.1; 376.6)	364.6 (345.8; 382.2)	374.1 (365.3; 382.7)	0.323
%NI	9.3 (7.6; 11.3)	9.8 (8.7; 11.0)	8.1 (6.6; 10.0)	9.7 (8.7; 10.7)	0.442
%NII	54.1 (51.3; 57.1)	54.5 (52.9; 56.2)	56.4 (53.5; 59.6)	53.9 (52.4; 55.3)	0.498
%NIII	17.7 (15.1; 20.3)	16.3 (14.8; 17.7)	16.3 (13.7; 19.0)	17.2 (15.9; 18.5)	0.690
%REM	17.6 (15.5; 19.7)	18.0 (16.8; 19.2)	18.4 (16.3; 20.5)	18.2 (17.2; 19.3)	0.946
SLEEP_WAKE	47.6 (37.7; 60.2)	44.2 (38.6; 50.5)	42.0 (33.1; 53.2)	43.3 (38.5; 48.7)	0.881
NARO	99.7 (80.5; 123.5)	119.3 (105.5; 134.9)	106.1 (85.4; 131.8)	112.4 (100.9; 125.2)	0.497
IND_NARO	19.4 (15.3; 24.0)	20.9 (18.4; 23.5)	17.7 (13.7; 22.2)	18.7 (16.7; 21.0)	0.527
RESP_BREAKS	104.4 (72.4; 150.7)	120.7 (97.8; 148.9)	89.5 (61.7; 129.7)	103.6 (86.2; 124.6)	0.517
APNEA	3.0 (1.7; 4.9)	2.5 (1.8; 3.3)	2.4 (1.3; 4.1)	2.7 (2.0; 3.5)	0.939
OBS_APNEA	1.7 (0.8; 3.0)	1.2 (0.8; 1.8)	1.2 (0.5; 2.3)	1.3 (0.9; 1.9)	0.852
CENT_APNEA	0.34 (0.04; 0.72)	0.61 (0.40; 0.86)	0.61 (0.25; 1.08)	0.60 (0.41; 0.82)	0.607
MIX_APNEA	0.23 (-0.01; 0.53)	0.26 (0.11; 0.43)	0.29 (0.03; 0.60)	0.28 (0.15; 0.43)	0.987
HYPOPNEA	74.2 (53.9; 101.9)	95.5 (79.6; 114.5)	71.4 (51.7; 98.5)	79.6 (67.9; 93.4)	0.279
SaO_Avg	93.3 (92.6; 94.0)	93.4 (93.0; 93.8)	93.4 (92.7; 94.1)	93.6 (93.3; 93.9)	0.790
SaO_Min	84.0 (81.7; 86.0)	83.6 (82.4; 84.9)	84.7 (82.5; 86.7)	84.4 (83.3; 85.4)	0.791
%SAO	1.2 (0.6; 1.9)	0.9 (0.6; 1.2)	0.6 (0.2; 1.1)	0.8 (0.6; 1.1)	0.516
AHI	20.3 (14.6; 27.0)	21.2 (17.7; 25.0)	16.8 (11.5; 23.1)	17.4 (14.6; 20.4)	0.344

Data are expressed as estimated averages (95% confidence interval); active (≥ 150 minutes) or inactive (< 150 minutes); short time (< 10 hours) or long time (≥ 10 hours). TTR = total recording time; SLEEP_LAT = sleep latency; REM_LAT = REM sleep latency; TTS = total sleep time, %NI = percentage sleep stage 1; %NII = percentage sleep stage 2; %NIII = percentage sleep stage III; %REM = percentage REM sleep; SLEEP_WAKE = awakening from sleep; NARO = nightly arousals; IND_NARO = nightly arousals index; RESP_BREAKS = respiratory breaks; OBS_APNEA = obstructive apnea; CENT_APNEA = central apnea; MIX_APNEA = mixed apnea; HYPOPNEA = hypopnea; SaO_Avg = average oxygen saturation; SaO_Min = minimum oxygen saturation; %SAO = percentage oxygen apnea; AHI = apnea-hypopnea index (events per hour).

### Logistic regression analysis: sitting time and screen time.

Multiple-approach logistic regression models were built in order to explain physical inactivity and sitting time (Table 5) or screen time (Table 6), with adjustments for age, gender and BMI. We found no evidence of association between physical inactivity and sleep quality (P > 0.05 in all analyses).

**Table 5.** Logistic regression model, evaluating the association between physical activity practice and sleep quality, controlled for age, gender, body mass index (BMI) and sitting time (6-hour and 10-hour cutoffs)

Variable	6 hours				Variable	10 hours			
	Physical activity		OR (95% CI)	P-value		Physical activity		OR (95% CI)	P-value
	Active (n = 203)	Inactive (n = 166)				Active (n = 203)	Inactive (n = 166)		
**Sleep quality (AHI)**					**Sleep Quality (AHI)**				
< 15 (n = 178)	107 (60.1%)	71 (39.9%)	0.892 (0.511; 1.558)	0.687	< 15 (n = 178)	107 (60.1%)	71 (39.9%)	0.919 (0.524; 1.609)	0.767
15-29.9 (n = 87)	40 (46.0%)	47 (54.0%)	1.468 (0.816; 2.641)	0.200	15-29.9 (n = 87)	40 (46.0%)	47 (54.0%)	1.477 (0.820; 2.662)	0.194
≥ 30 (n = 104)	56 (53.8%)	48 (46.2%)	reference	---	≥ 30 (n = 104)	56 (53.8%)	48 (46.2%)	reference	---
**Age (years)**					**Age (years)**				
Average (SD)	45.4 (12.5)	44.7 (11.7)	0.991 (0.973; 1.009)	0.331	Average (SD)	45.4 (12.5)	44.7 (11.7)	0.990 (0.972; 1.008)	0.276
**Gender**					**Gender**				
Female (n = 122)	73 (59.8%)	49 (40.2%)	0.849 (0.529; 1.362)	0.497	Female (n = 122)	73 (59.8%)	49 (40.2%)	0.802 (0.495; 1.300)	0.371
Male (n = 247)	130 (52.6%)	117 (47.4%)	reference	---	Male (n = 247)	130 (52.6%)	117 (47.4%)	reference	---
**BMI (kg/m^2^)**					**BMI (kg/m^2^)**				
Average (SD)	28.1 (4.9)	29.1 (5.3)	1.035 (0.989; 1.083)	0.140	Average (SD)	28.1 (4.9)	29.1 (5.3)	1.038 (0.992; 1.087)	0.106
**Sitting time**					**Sitting time**				
< 6 h (n = 18)	8 (44.4%)	10 (55.6%)	1.822 (0.684; 4.855)	0.230	< 10 h (n = 81)	40 (49.4%)	41 (50.6%)	1.582 (0.933; 2.684)	0.089
≥ 6 h (n = 351)	195 (55.6%)	156 (44.4%)	reference	---	≥ 10 h (n = 288)	163 (56.6%)	125 (43.4%)	reference	---

OR = odds ratio; CI = confidence interval; SD = standard deviation; n = number; AHI = apnea-hypopnea index (events per hour).

**Table 6.** Logistic regression model. evaluating the association between physical activity practice and sleep quality, controlled for age, gender, body mass index (BMI) and screen time (6-hour and 10-hour cutoffs).

6 hours					10 hours				
Variable	Physical activity		OR (95% CI)	P-value	Variable	Physical Activity		OR (95% CI)	P-value
	Active (n = 203)	Inactive (n = 166)				Active (n = 203)	Inactive (n = 166)		
**Sleep quality (AHI)**					**Sleep quality (AHI)**				
< 15 (n = 178)	107 (60.1%)	71 (39.9%)	0.902 (0.516; 1.578)	0.718	< 15 (n = 178)	107 (60.1%)	71 (39.9%)	0.881 (0.505; 1.538)	0.656
15 - 29.9 (n = 87)	40 (46.0%)	47 (54.0%)	1.473 (0.819; 2.651)	0.196	15 - 29.9 (n = 87)	40 (46.0%)	47 (54.0%)	1.469 (0.818; 2.640)	0.198
≥ 30 (n = 104)	56 (53.8%)	48 (46.2%)	reference	---	≥ 30 (n = 104)	56 (53.8%)	48 (46.2%)	Reference	---
**Age (years)**					**Age (years)**				
Average (SD)	45.4 (12.5)	44.7 (11.7)	0.990 (0.972; 1.008)	0.273	Average (SD)	45.4 (12.5)	44.7 (11.7)	0.994 (0.975; 1.012)	0.498
**Gender**					**Gender**				
Female (n = 122)	73 (59.8%)	49 (40.2%)	0.817 (0.505; 1.322)	0.410	Female (n = 122)	73 (59.8%)	49 (40.2%)	0.891 (0.554; 1.431)	0.632
Male (n = 247)	130 (52.6%)	117 (47.4%)	reference	---	Male (n = 247)	130 (52.6%)	117 (47.4%)	Reference	---
**BMI (kg/m^2^)**					**BMI (kg/m^2^)**				
Average (SD)	28.1 (4.9)	29.1 (5.3)	1.038 (0.991; 1.086)	0.115	Average (SD)	28.1 (4.9)	29.1 (5.3)	1.031 (0.985; 1.079)	0.194
**Screen time**					**Screen time**				
< 6 hours (n = 63)	32 (50.8%)	31 (49.2%)	1.506 (0.838; 2.705)	0.171	< 10 hours (n = 156)	91 (58.3%)	65 (41.7%)	0.899 (0.571; 1.414)	0.644
≥ 6 hours (n = 306)	171 (55.9%)	135 (44.1%)	reference	---	≥ 10 hours (n = 213)	112 (52.6%)	101 (47.4%)	reference	---

OR = odds ratio; CI = confidence interval; SD = standard deviation; n = number; AHI = apnea-hypopnea index (events per hour).

## DISCUSSION

Our study showed a correlation between sleep quality and SED and was concordant with the findings from a previous study.^14^ In this study, we used PSG indicators to outline the results. We identified correlations between sitting time and hypopnea and between screen time and hypopnea. In addition, we observed a negative correlation between average oxygen saturation and total physical activity time.

Further to this, we identified that individuals with suspected sleep disorders who were inactive and had short screen time (< 6 hours) had a lower percentage of REM sleep than did individuals with suspected sleep disorders who were inactive and had long screen time (≥ 6). We also showed that awakening from sleep occurred more among individuals with suspected sleep disorders who were inactive and had short screen time (< 6 hours) than among individuals with suspected sleep disorders who were active and had long screen time (≥ 6 hours). This had also been demonstrated in a previous study.^14^ Therefore, screen time may be associated with a decrease in sleep quality.

The main finding of our study was that after adjusting for anthropometric and clinical factors, SED analyzed in terms of sitting time and screen time was not associated with sleep quality. Previous studies also suggested that OSA was affected by age, because the prevalence of OSA increased up to the age of 65 years, at which point, for unclear reasons, the prevalence reached a threshold. Previous data also suggested the interaction between body weight, BMI and OSA in elderly people may be different to that of young adults. Therefore, obesity predisposes and potentiates OSA.^26^ In this regard, the prevalence among obese or severely obese patients is almost twice that of normal obese adults. In addition, patients with moderate OSA who gain 10% of their baseline weight present a sixfold increased risk of OSA progression. However, individuals who reduce the same percentage of weight can present an improvement of 20% in the severity of OSA.^26^

It is possible that obesity may worsen OSA due to fat deposition at specific sites. Deposition of fat in the tissues surrounding the upper airways seems to result in a lower lumen and greater collapsibility of the upper airways, thus predisposing to apnea.^27,28^ In addition, fat deposits around the thorax (truncal obesity) reduce thoracic complacency and functional residual capacity and may increase the demand for oxygen.^29^

In this sense, visceral obesity is also considered to be a risk factor for OSA. However, the relationship between OSA and visceral obesity is complex. Although there is evidence showing obesity, as well as visceral obesity, may predispose to OSA and that weight loss results in OSA improvement, previous studies have suggested that OSA may itself cause weight gain.^30,31^ Some anthropometric indices, including waist circumference, are widely used as markers for obesity or central obesity.^32^ In a recent study, a 1 cm increase in waist circumference gave rise to an 11% increase in the risk of development of OSA.^33^

The prevalence of OSA varies according to gender: it is approximately 30% in men and 15% in women. Mechanisms that potentially explain gender differences in the prevalence and severity of OSA include significant variation in body fat distribution, upper airway collapsibility, hormonal status and ventilatory control.^34,35,36^

In addition, OSA is a recognized cause of secondary hypertension.^37^ Episodes of OSA impose multiple injury; however, intermittent hypoxia (rather than hypercapnia, sleep disruptions or intrathoracic pressure oscillations) is thought to be the most important prohypertensive.^37^ Although the mechanisms underlying OSA-related hypertension are not fully understood, the current concept suggests that the sympathetic nervous system and the renin-angiotensin system alter vascular function and structure, resulting in blood pressure elevation. Sympathetic nervous system activity during sleep and wakefulness is heightened in patients with OSA. The mechanisms that sustain sympathetic activation after withdrawal of chemical stimuli are not known; however, it appears that this chronic sympathetic excitation has both reflex and central nervous system origins.^37^

Sitting time and screen time have been associated with less favorable serum biomarkers, besides being considered to be new risk factors for chronic diseases, including cardiovascular diseases, diabetes and cancers, and new risk factors for a higher mortality rate, regardless of PA levels. In contrast to a previous study^14^ that showed that SED presented a high risk of severe OSA, in our study it was not associated with moderate or severe OSA after adjusting for clinical factors (BMI, gender and age). One possible explanation for our findings relates to the sample composition and the diagnostic method used for OSA.

Previous studies did not investigate the association between PA levels and OSA in populations.^14,38^ One study only used a female sample.^38^ Our study was composed of individuals of both genders who visited the neurophysiology clinic, with a referral to undergo overnight laboratory polysomnography due to suspected sleep disorders. The data presented in our study and previous studies^14,38^ enable us to hypothesize that populations with suspected sleep disorders can influence the results demonstrated, if anthropometric data such as BMI are different. Polysomnography is considered to be the gold-standard method for evaluating OSA, and the diagnoses thus obtained reinforce our findings. A recent study^14^ that investigated the association between screen time and the risk of OSA used a questionnaire to diagnose the risk of developing this condition.

Screen time (predominantly seated leisure time) has been consistently correlated with adverse health outcomes. A recent study demonstrated the association between screen time and OSA^14^ and indicated that screen time may be an important risk factor for sleep disorders and the risk of apnea. In our study, associations between screen time per week or weekend and OSA were observed only through correlations. We failed to find any clear explanation for these results. One possibility would be to consider that there may have been errors in completing questionnaires, which was also suggested in another recent study,^38^ because of individuals’ altered perceptions of screen time. In a study that identified an association between screen times and OSA risk, it was speculated that the main factor could be the proximity of television viewing to going to sleep,^14^ thus assuming that screen time would happen at night, close to bedtime. We emphasize that the sleep measurements in this study were not evaluated using questionnaires, especially when checking the risk of OSA. Questionnaires cannot diagnose OSA and can introduce errors into the results obtained. Our study used the gold standard method to assess OSA, which therefore strengthens the findings obtained.

In relation to PA levels, experimental and intervention studies support the notion that there is a bi-directional relationship between sleep and PA.^38^ However, these studies do not necessarily provide insight into sleep and PA patterns. From a clinical standpoint there is growing evidence that aerobic exercise training could be beneficial for adults with a diagnosed sleep disorder.^39^

Results from previous studies have indicated that men and women with low PA levels have the highest odds of OSA.^40^ Furthermore, there seems to be an inverse relationship between PA level and OSA severity.^41^ Reduced PA is associated with increased OSA severity, independent of gender, age and BMI.^41,42^ These findings also indicate that efforts to prevent OSA should include encouraging patients to engage in at least some form of moderate-to-vigorous PA.^43^ In addition, findings regarding PA may be different among patients with a wide distribution of OSA severity. The reasons for limiting exercise among patients with OSA are unclear. Some potential contributing factors comprise dyspnea, muscle weakness in the lower limbs, cardiac dysfunction, respiratory muscle dysfunction, arterial hypoxemia, demotivation and peripheral vascular diseases.^39,44^

The strengths of the current study included, first, its use of PSG for diagnosing OSA. Second, it used total sitting time and total screen time per week and on weekends. Third, it incorporated information on BMI, age, gender, waist circumference, AH presence and PA level, which allowed us to limit the effects of confounding variables. It is possible, however, that additional confounding effects may have been present.

The limitations of the current study included, first, the cross-sectional nature of this study, which limited the effects of causal inferences. A second limitation related to the sample size, which meant that only a specific population could be analyzed. Lastly, other limitations related to the administration of self-reported measurements of PA and SED levels, and lack of investigation of the specific periods during which individuals remained in front of screens. In a general manner, the present study may serve to generate hypotheses for future research. Future studies should longitudinally investigate the associations between sitting time, screen time, PA and sleep.

## CONCLUSIONS

We did not identify any relationship between screen time and sitting time and OSA among adults with suspected sleep disorders, after adjusting for anthropometric and clinical factors.
